# Complete Genome Sequence of *Sulfurospirillum* sp. Strain ACS_DCE_, an Anaerobic Bacterium That Respires Tetrachloroethene under Acidic pH Conditions

**DOI:** 10.1128/MRA.01360-20

**Published:** 2021-01-14

**Authors:** Yi Yang, Leitao Huo, Xiuying Li, Jun Yan, Frank E. Löffler

**Affiliations:** a Key Laboratory of Pollution Ecology and Environmental Engineering, Institute of Applied Ecology, Chinese Academy of Sciences, Shenyang, Liaoning, China; b University of Chinese Academy of Sciences, Beijing, China; c Center for Environmental Biotechnology, University of Tennessee, Knoxville, Tennessee, USA; d Department of Microbiology, University of Tennessee, Knoxville, Tennessee, USA; e Department of Civil and Environmental Engineering, University of Tennessee, Knoxville, Tennessee, USA; f Department of Biosystems Engineering & Soil Science, University of Tennessee, Knoxville, Tennessee, USA; g Biosciences Division, Oak Ridge National Laboratory, Oak Ridge, Tennessee, USA; h Joint Institute for Biological Sciences (JIBS), Oak Ridge National Laboratory, Oak Ridge, Tennessee, USA; University of Southern California

## Abstract

*Sulfurospirillum* sp. strain ACS_DCE_ couples growth with reductive dechlorination of tetrachloroethene to *cis*-1,2-dichloroethene at pH values as low as 5.5. The genome sequence of strain ACS_DCE_ consists of a circular 2,737,849-bp chromosome and a 39,868-bp plasmid and carries 2,737 protein-coding sequences, including two reductive dehalogenase genes.

## ANNOUNCEMENT

Bioremediation of chlorinated contaminants in acidic groundwater is challenging because most organohalide-respiring bacteria cease growth under acidic conditions (1–3). *Sulfurospirillum* strains that grow via tetrachloroethene (PCE) reductive dechlorination under acidic conditions are candidates for bioremediation of chlorinated contaminants in low-pH groundwater (2, 4). *Sulfurospirillum* sp. strain ACS_DCE_ was isolated from a PCE-dechlorinating microcosm established with contaminated soil collected at the Axton Cross Superfund site (Holliston, MA, USA) (2). Strain ACS_DCE_ couples growth with PCE-to-*cis*-1,2-dichloroethene (*c*DCE) reductive dechlorination at pH values as low as 5.5 (2) and shares 99.3% and 98.5% 16S rRNA gene sequence identities with the PCE-to-trichloroethene dechlorinating *Sulfurospirillum* sp. strain ACS_TCE_ (GenBank accession number CP045453.2) (5) and the PCE-to-*c*DCE dechlorinating Sulfurospirillum multivorans strain DSM 12446 (CP007201.1) (4), respectively.

Strain ACS_DCE_ was grown in anoxic, 2-(*N*-morpholino)-ethanesulfonic acid-buffered mineral salt medium (pH 5.5) with acetate as the carbon source, hydrogen as the electron donor, and PCE as the electron acceptor (2). Cells were collected from a 2-liter culture by centrifugation, and DNA was extracted using the cetyltrimethylammonium bromide method (6). Genome sequencing used a hybrid approach incorporating the Illumina and PacBio platforms. For Illumina sequencing, a library with an average insert size of 350 bp was constructed using the TruSeq DNA sample preparation kit (Illumina, San Diego, CA, USA), and paired-end sequencing (2 × 150 bp) was performed on an Illumina HiSeq 2000 instrument. Adaptors and low-quality sequences were removed from the raw reads using Cutadapt version 1.9.1 (7). For PacBio sequencing (PacBio RS II/Sequel SMRT), genomic DNA was sheared using g-TUBEs (Covaris, Inc., Woburn, WA, USA) to generate 10-kb fragments, followed by ligation with universal hairpin adapters using the SMRTbell template prep kit version 1.0 (Pacific Biosciences, Menlo Park, CA, USA) (8). The PacBio raw read *N*_50_ value was 3,932 bp. Genome assembly with 273,566 PacBio raw long reads (coverage, 300×) using Flye version 2.6 (9) was refined with 25,162,740 Illumina short reads (coverage, 1,387×) using Pilon version 1.20.1 (10). The assembly resulted in a 2,737,849-bp circular chromosome with a G+C content of 38.8% and a 39,868-bp circular plasmid with a G+C content of 35.6%. Circlator version 1.5.1 (11) was used to remove the overlapping ends, circularize the genome, and rotate the chromosomal origin of replication to the starting position of the *dnaA* gene. Gene identification and functional annotation were performed using the NCBI Prokaryotic Genome Annotation Pipeline version 4.12 (12). Default parameters were used for all software unless otherwise specified.

The strain ACS_DCE_ chromosome contains 2,737 protein-coding sequences, 47 tRNAs, and 3 rRNA operons organized in the order 16S, 23S, 5S. The chromosome harbors two putative reductive dehalogenase (*rdh*) A genes (FA584_00035 and FA584_00060), both adjacent to a downstream *rdhB* gene encoding a membrane anchor protein. The strain ACS_DCE_ chromosome harbors a fumarate reductase gene cluster (FA584_09715, FA584_09720, and FA584_09725) and a gene (FA584_11415) coding for the catalytic subunit NapA of periplasmic nitrate reductase, suggesting that nitrate and fumarate are alternate electron acceptors. The plasmid harbors 47 genes, including genes encoding transposases (four), DNA polymerase IV (two), and subunits of type II toxin-antitoxin systems (six). The results expand the *Sulfurospirillum* pangenome and identify biomarkers for monitoring reductive dechlorination activity in low-pH groundwater.

### Data availability.

The genome project and sample are indexed at GenBank under BioProject PRJNA534159 and BioSample SAMN11478956. The GenBank accession numbers of the chromosomal and plasmid sequences are CP039734 and CP059996, respectively. The raw sequencing reads have been deposited in the Sequence Read Archive (SRA) under accession numbers SRR12322185 (Illumina) and SRR12322184 (PacBio).

## References

[1] Henterly RW, Harms WD 2015 Optimal treatment zone moves during enhanced reductive dechlorination in fractured bedrock. Remediation 25:81–88. doi:10.1002/rem.21441.

[2] Yang Y, Cápiro NL, Marcet TE, Yan J, Pennell KD, Löffler FE 2017 Organohalide respiration with chlorinated ethenes under low pH conditions. Environ Sci Technol 51:8579–8588. doi:10.1021/acs.est.7b01510. 28665587

[3] Yang Y, Cápiro NL, Yan J, Marcet TF, Pennell KD, Löffler FE 2017 Resilience and recovery of *Dehalococcoides mccartyi* following low pH exposure. FEMS Microbiol Ecol 93:fix130. doi:10.1093/femsec/fix130. 29040515

[4] Scholz-Muramatsu H, Neumann A, Meßmer M, Moore E, Diekert G 1995 Isolation and characterization of *Dehalospirillum multivorans* gen. nov., sp. nov., a tetrachloroethene-utilizing, strictly anaerobic bacterium. Arch Microbiol 163:48–56. doi:10.1007/BF00262203.

[5] Huo L, Yang Y, Lv Y, Li X, Löffler FE, Yan J 2020 Complete genome sequence of *Sulfurospirillum* strain ACS_TCE_, a tetrachloroethene-respiring anaerobe isolated from contaminated soil. Microbiol Resour Announc 9:e00941-20. doi:10.1128/MRA.00941-20. 33004458 PMC7530930

[6] Joint Genome Institute. 2012 Bacterial genomic DNA isolation using CTAB. http://jgi.doe.gov/wp-content/uploads/2014/02/JGI-Bacterial-DNA-isolation-CTAB-Protocol-2012.pdf.

[7] Martin M 2011 Cutadapt removes adapter sequences from high-throughput sequencing reads. EMBnet J 17:10–12. doi:10.14806/ej.17.1.200.

[8] McCarthy A 2010 Third generation DNA sequencing: Pacific Biosciences' single molecule real time technology. Chem Biol 17:675–676. doi:10.1016/j.chembiol.2010.07.004. 20659677

[9] Lin Y, Yuan J, Kolmogorov M, Shen MW, Chaisson M, Pevzner PA 2016 Assembly of long error-prone reads using de Bruijn graphs. Proc Natl Acad Sci U S A 113:E8396–E8405. doi:10.1073/pnas.1604560113. 27956617 PMC5206522

[10] Walker BJ, Abeel T, Shea T, Priest M, Abouelliel A, Sakthikumar S, Cuomo CA, Zeng Q, Wortman J, Young SK, Earl AM 2014 Pilon: an integrated tool for comprehensive microbial variant detection and genome assembly improvement. Plos One 9:e112963. doi:10.1371/journal.pone.0112963. 25409509 PMC4237348

[11] Hunt M, De Silva N, Otto TD, Parkhill J, Keane JA, Harris SR 2015 Circlator: automated circularization of genome assemblies using long sequencing reads. Genome Biol 16:294. doi:10.1186/s13059-015-0849-0. 26714481 PMC4699355

[12] Tatusova T, DiCuccio M, Badretdin A, Chetvernin V, Nawrocki EP, Zaslavsky L, Lomsadze A, Pruitt KD, Borodovsky M, Ostell J 2016 NCBI Prokaryotic Genome Annotation Pipeline. Nucleic Acids Res 44:6614–6624. doi:10.1093/nar/gkw569. 27342282 PMC5001611

[13] Jalili V, Afgan E, Gu Q, Clements D, Blankenberg D, Goecks J, Taylor J, Nekrutenko A 2020 The Galaxy platform for accessible, reproducible and collaborative biomedical analyses: 2020 update. Nucleic Acids Res 48:W395–W402. doi:10.1093/nar/gkaa434. 32479607 PMC7319590

